# The clinical outcome of intracranial hemangioblastomas treated with linac-based stereotactic radiosurgery and radiotherapy

**DOI:** 10.1093/jrr/rrt235

**Published:** 2014-02-18

**Authors:** Putipun Puataweepong, Mantana Dhanachai, Ake Hansasuta, Somjai Dangprasert, Chomporn Sitathanee, Parmon Puddhikarant, Chuleeporn Jiarpinitnun, Rawee Ruangkanchanasetr, Patchareporn Dechsupa, Kumutinee Pairat

**Affiliations:** 1 Radiation and Oncology Unit, Department of Radiology, Faculty of Medicine, Ramathibodi Hospital, Mahidol University, 270, Rama VI Road, Ratchathewi, Bangkok, Thailand; 2 Department of Surgery, Faculty of Medicine, Ramathibodi Hospital, Mahidol University, 270, Rama VI Road, Ratchathewi, Bangkok 10400, Thailand; 3 Radiosurgery Center, Faculty of Medicine, Ramathibodi Hospital, Mahidol University, 270, Rama VI Road, Ratchathewi, Bangkok 10400, Thailand

**Keywords:** Hemangioblastoma, radiosurgery, stereotactic radiotherapy, X-Knife, CyberKnife

## Abstract

Recent publications have reported stereotactic radiosurgery as an effective and safe treatment for intracranial hemangioblastomas. However, because of the low incidence of these particular tumors, reports on large patient number studies have not yet been available. The objective of this study was to analyze the clinical results of 14 patients with 56 intracranial hemangioblastomas treated with linear accelerator (linac)-based stereotactic radiosurgery (SRS) and radiotherapy (SRT) in the same institute. The median age of patients was 41 years (range, 28–73 years). Nine of the patients (64%) had von Hippel-Lindau disease. A total of 39 lesions (70%) were treated with CyberKnife (CK), and 17 lesions (30%) were treated with X-Knife. The median pretreatment volume was 0.26 cm^3^ (range, 0.026–20.4 cm^3^). The median marginal dose was 20 Gy (range, 10–32 Gy) in 1 fraction (range, 1–10 fractions). The median follow-up time was 24 months (range, 11–89 months). At the last follow-up, 47 tumors (84%) were stable, 7 (13%) decreased and 2 (4%) increased. The 1-, 2- and 6-year local control rates were 98%, 88% and 73%, respectively. No radiation complications were observed in this study. There was a trend toward local failure only in cystic tumors, but this trend was not found to be statistically significant. SRS/SRT achieved a high local control rate in intracranial hemangioblastomas without radiation-induced complications.

## INTRODUCTION

Hemangioblastomas (HBs) are rare tumors of the central nervous system (CNS). According to the 2007 WHO classification, the origin of HBs remains enigmatic. Their most common CNS locations include the cerebellum, brainstem and spinal cord. These tumors account for 1–2% of primary CNS tumors in adults [1]. Hemangioblastomas can be found as single tumors or, in ∼10%, as multiple tumors, as in those associated with von Hippel-Lindau (VHL) disease. Surgical resection has been established as the effective treatment of choice for hemangioblastomas. However, the surgical option is limited in patients with either multiple CNS tumors or a deep-seated location, as these characteristics may result in unacceptable surgical risk. Stereotactic radiosurgery (SRS) and radiotherapy (SRT) have their roles in the treatment for CNS hemangioblastomas, either as a sole modality of treatment or as a post-surgery adjuvant for residual or recurrent lesions. Because of the low incidence of this particular type of tumor, reports on large patient number studies have not yet been available. Hence, the objective of this study was to analyze the clinical outcome for hemangioblastoma patients treated with both frame-based linac radiosurgery (X-Knife) and frameless robotic radiosurgery (CyberKnife, CK), both in terms of local control and complications associated with the treatment at one institution.

## MATERIALS AND METHODS

### Patients

This study was approved by the institutional ethics committee. Informed consent was obtained from all patients. Before treatment, the management of patients was discussed in the radiosurgery board meeting. Eligibility criteria for treatment included the following: (i) a newly diagnosed tumor with interval growth on serial MRI scans; (ii) a postoperative residual or recurrent tumor; (iii) surgical or medical contraindication to surgery; and/or (iv) patient preference. Most of the patients were treated with SRS, except for those with a large tumor (>3 cm) or a tumor that was located close to the optic apparatus and brain stem, in which case they were treated with hypofraction stereotactic radiotherapy (HSRT).

Between 1998 and 2010, 56 hemangioblastomas in 14 patients were treated with linac-based radiosurgery at the radiosurgery center, Ramathibodi Hospital. All patients were followed up prospectively until death. There were 10 males (71%) and 4 females (29%), with a median age of 41 years (range, 28–73 years). The median pretreatment Karnofsky performance status (KPS) was 90 (range, 100–70). VHL disease was found in 9 patients (64%), while the other 5 had sporadic hemangioblastomas. The presenting symptoms included cerebellar signs (13), visual deficit (1) and cranial nerve paresis (1). Location of the tumors were as follows: cerebellum (*n* = 44, 79%), brainstem (*n* = 9, 16%), and optic chiasm (*n* = 3, 5%). Of the 14 patients, 13 underwent surgical resection before SRS/SRT. SRS was given as the sole modality in 1 patient with 10 lesions. Eight patients had multiple tumors, and 6 had a single lesion. The median number of lesion was 3 (range, 1–10).

### Radiation technique

#### Frame-based linac radiosurgery (X-Knife)

In 1997, the Radiosurgery Center at Ramathibodi Hospital established the first dedicated linac-based stereotactic radiation machine in Thailand. The system included a 6-MV dedicated linac with fixed circular cones (Varian); this was used with the X-Knife forward-planning system, versions 3 & 4 (Radionics). For a patient immobilization and positioning system, the Brown–Robert–Wells (BRW) stereotactic head frame was applied (with the assistance of a neurosurgeon) for the SRS technique, while the relocatable Gill–Thomas–Cosman frame was used for the HSRT technique. A collimator size that covered ≥ 90% of the target volume was selected. Multiple isocenters were used in irregularly shaped targets. High conformity was established by using a non-coplanar arc with different beam weighting.

#### Frameless robotic radiosurgery (CK)

In 2009, the first robotic radiosurgery (CK) in Thailand became operational at our hospital. The CK model G4 (Accuray Inc., Sunnyvale, CA, USA) uses a 6-MV light-weight linac mounted on a fully articulated robotic arm. Multiplan (Accuray) software was used for inverse planning. Patients were immobilized in the supine position with a thermoplastic facemask.

Individual treatment planning was done at a workstation using an image set from a contrast-enhanced CT scan, 1.25 mm-slice thickness, with or without gadolinium-enhanced MRI. Target and critical organ contouring was done by physicians, and a treatment plan was generated by medical physicist. Gross tumor volume (GTV) and critical structures were contoured in each consecutive slice of CT and MRI. No additional margin was added to the GTV to obtain the planning target volume (PTV).

Dose prescription, in general, was determined by the volume of the tumor and of the normal tissue exposed to radiation. A single-fraction dose for HB in our study typically ranged from 11–15 Gy. If a single fraction plan would expose critical structures to radiation beyond their tolerance, such as 10–12 Gy for the brainstem or optic pathway, HSRT of 2–5 fractions was considered.

The prescribed radiation dose was delivered to the isodose surface that covers, ideally, >95% of the target volume. Finally, treatment planning was evaluated by our radiosurgery team (consisting of a neurosurgeon, a radiation oncologist and a medical physicist).

In the first period, prior to CK installation (1997–2009), four patients with 17 (30%) lesions were treated with X-Knife. After CK installation, the next 10 patients with 39 (70%) lesions were treated with CK. The change from X-Knife to CK was primarily based on CK's superior availability to the non-invasive frame applied to the patient. A total of 38 lesions (68%) were treated with SRS, and 18 lesions (32%) were treated with HSRT. The median marginal dose of X-Knife was 21 Gy (range, 10–32 Gy) in 1 fraction (range, 1–10 fractions) and of CK was 20 Gy (range, 11.5–30 Gy) in 1 fraction (range, 1–5 fractions). The median pretreatment tumor volume was 0.26 cm^3^ (range, 0.026–20.4 cm^3^). The details of treatment are summarized in Table 1.

**Table 1.** Treatment characteristics of 14 patients with 56 lesions

parameter	value
Treatment machine (number of lesions) (%)	
X-Knife	17 (30)
CK	39 (70)
Radiation technique (number of lesions) (%)	
SRS	38 (68)
HSRT	18 (32)
Median marginal dose (Gy) (range)	
X-Knife	21 (10–32)
CK	20 (11.5–30)
Median number of fractions (range)	
X-Knife	1 (1–10)
CK	1 (1–5)
Median prescribed isodose (%) (range)	
X-Knife	70 (54–81)
CK	80 (60–90)
Median pretreatment tumor volume (cm^3^) (range)	0.26 (0.026–20.4)

CK = CyberKnife, SRS = stereotactic radiosurgery, HSRT = hypofraction stereotactic radiotherapy.

### Post treatment clinical evaluation

After treatment, all patients were assessed clinically and radiologically at routine intervals of follow-up. Response to treatment was evaluated using follow-up MRI, and the outcomes were classified as follows: (i) complete response: complete disappearance of tumor; (ii) partial response: decrease in tumor size of > 15%; (iii) stable disease: no obvious change in tumor size; and (iv) progression: increase in tumor size of > 15% [2].

### Statistical analyses

Continuous variables are presented as median and range. Categorical variables are presented with frequency and percentage. The primary endpoint, the local control (LC) rate, was defined as the time from the date of start SRS/HSRT until local tumor progression (detected from clinical examination or imaging study) or the date of last follow-up. Complete response, partial response and stable disease (from imaging study) were defined as tumor control. Adverse events were graded according to the Common Terminology Criteria for Adverse Events, version 3.0. The survival probability was calculated using Kaplan–Meier methods and compared using the log-rank test. Multivariate analysis was done using the Cox proportional hazard model. All statistical analyses were performed using SPSS software, version 16.0.

## RESULTS

**Fig. 1.**
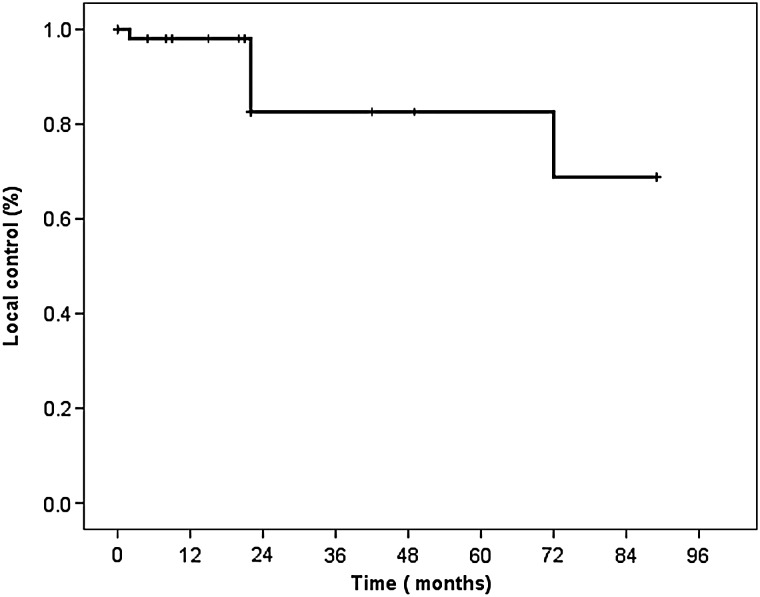
Local control of 56 lesions of hemangioblastomas treated with linac-based SRS and SRT. The 1-, 2- and 6-year local control rate was 98%, 88% and 73%, respectively.

The median follow-up time was 24 months (range, 11–89 months). The details of demographic, radiosurgical and follow-up data for the 14 patients are summarized in Table 2. At the most recent follow-up, one patient had died from renal cell carcinoma without evidence of intracranial progression. Two tumors (4%) presented as increased in volume, 47 (84%) were stable in size, and 7 (13%) decreased. The 1-, 2- and 6-year LC rates for all patients were 98%, 88% and 73%, respectively (Fig. 1). In two patients who had tumor progression, there was enlargement of the cystic portion of the tumor; these patients were treated with salvage surgery. No significant factor associated with the LC rate was found after univariate testing.

**Table 2.** Demographic and radiosurgical data of 14 patients with 56 lesions

Pt	No. of lesions	Machine	Tech/no of lesions	Dose (marg./max) (Gy)	No of Fx.	tumor volume ( cm^3^)		F/U (months)
						Before treatment	At last F/U	
1	1	CK	SRS/1	14/25	1	8.5 Cerebellum	17.16 cm^3^ Progression	11
2	8	CK	SRS/8	11.5/19–21 13/18.5–21 14/19	1 1 1	0.027, 0.033, 0.058, 0.11 0.13, 0.25, 1.05 0.33 Cerebellum	PR	15
3	3	CK	HSRT/3	21.25/28–32	5	0.048, 0.056, 0.895 Brainstem	SD	14
4	7	CK	SRS/5 HSRT/2	20/25–30 24/28–23	1 3	0.026, 0.028, 0.051, 0.069, 0.109 Cerebellum 0.09, 0.14 Brainstem	SD	27
5	1	CK	HSRT/1	22.5/25	5	20.4 Optic chiasm	SD	24
6	1	CK	SRS/1	20/28	1	0.26 Cerebellum	SD	20
7	4	CK	SRS/4	20/24	1	0.1, 0.19, 0.37, 0.58 Cerebellum	SD	12
8	6	CK	HSRT/6	30/38 25/34–39	3 5	3.19, 4.6 Cerebellum 0.28, 0.31, 0.34, 0.87 Cerebellum	SD	12
9	1	CK	HSRT/1	25/34	5	15.69 Optic chiasm	SD	14
10	7	CK	SRS/4 HSRT/3	14/19–20 20/28	1 5	0.066, 0.144, 0.217, 0.28 Cerebellum 0.41, 0.71, 1.26 Brain stem	SD	24
11	5	linac	SRS/5	10/13–17	1	0.2, 0.26, 1.14, 2.64, 6.08 Cerebellum	SD	42
12	10	Linac	SRS/10	12/17 14/19 15/20 16/19 20/25	1	0.24, 0.57 0.04, 0.05, 0.06 0.08, 0.11 0.3 1.75, 3 Cerebellum	SD	89
13	1	Linac	HSRT/1	30/41	10	2 Optic chiasm	SD	72
14	1	Linac	HSRT/1	32/36	10	7.65 Brainstem	12.56 cm^3^ Progression	49

SRS = stereotactic radiosurgery, HSRT = hypofraction stereotactic radiotherapy, PR = partial response, SD = stable disease.

Patients were found to tolerate treatment very well. There was no need for any premedication before or during treatment. No other severe radiation complication was observed in this study.

## DISCUSSION

Radiotherapy has proven its efficacy and safety for CNS hemangioblastoma treatment for over two decades [3–20]. Fractionated conventional external beam radiation therapy (EBRT) has a well-established role in the management of various intracranial and/or spinal cord diseases [3, 4]. Although conventional EBRT data have indicated a similar success rate in tumor control with a longer period of follow-up compared with advanced radiation technology, the desire to avoid unnecessary normal CNS tissue exposure to radiation makes it comparatively less attractive. In addition, with 4–6 weeks of total treatment, patient inconvenience and the expenses of a daily commute have to be taken into consideration. The cost saved by avoiding daily transportation to and from the radiotherapy facility can be perceived as significant by patients who choose the single or hypofractionated SRS regimen.

Advanced radiation technology utilizes the stereotactic concept to deliver an impressively accurate, highly conformal and large radiation dose to a target, while limiting beam exposure for nearby critical structures. For single-fraction treatment (SRS), the treatment targets should ideally be small (<30 mm) because of the dose–volume-dependent risk of delayed radiation injury. In contrast, fractionated SRT, using a relocatable head frame, allows larger treatment volume.

Our radiosurgery center possesses both frame-based radiosurgery and frameless radiosurgery in order to provide treatments for patients with a wide variety of tumor types. Because of the advantages of the CK over X-Knife in frameless immobilization during treatment and its ability to accurately deliver radiotherapy to extracranial targets, our department has transferred the majority of the patients for stereotactic irradiation to the CK unit. However, the X-Knife has been maintained because of its ability to fractionate treatment, as in conventional EBRT.

### Tumor control

Our results showed that the overall tumor control rate was 98%, 88% and 73% at 1, 2 and 6 years, respectively. For the short-term outcome, our results were comparable with those of previous reports, which ranged from 85–100% [5–16]. However, for the long-term follow-up, our result indicated 73% LC at 6 years. This falls in the lower range of the literature, where the comparable rates were between 71 and 100% [5–16]. The difference in the LC between the previous studies might be due to a difference in the definition of tumor control. For example, our study and Sayer *et al.* [15] had similar tumor control definitions that considered the change in sizes of both solid and cystic components. Therefore, our result of 73% LC at 6 years was comparable with the 5-year LC rate of 71% reported by Sayer *et al.*, in contrast to Tago *et al.* [9], who reported the 5-year LC rate as 96%. This high LC rate might be due to the fact that the authors defined tumor control as lack of enlargement of the solid component on imaging, regardless of any change in size of the cystic component. Table 3 summarizes the LC rates from previous reports of intracranial and spinal hemangioblastomas treated with radiosurgery.

**Table 3.** Summary of the local control rates from the previously reported series of intracranial and spinal hemangioblastoma treated with radiosurgery

author	No of Pts	Location	RT technique	Sporadic/VHL	Median F/U (range) (months)	Median dose (range) (Gy)	Median volume (range) (cm^3^)	2 year	5 year	10 year	Complication
Niemela, 1996 [5]	10	brain	GK	6/4	26 (4–68)	6–25 (20–25) 7–10 (5–19)	N/A		100		1 Cerebellar edema 1 SIADH
Patrice, 1996 [6]	22	brain	linac/GK	13/9	24.5 (6–77)	15.5 (12–20)	0.97 (0.05–12)	86			none
Jawahar, 2000 [7]	27	brain	GK	13/14	48 (6–108)	16 (11.7–20)	3.2 (0.36–27)	89.5	75.2		none
Park, 2005 [8]	9	brain	GK	4/5	51 (8.6–141)	16.6 (12.8–29.75)	N/A	96			1-radiation-induced brain injury
Tago, 2005 [9]	13	brain	GK	6/7	36 (3–159)	20 (18–20)	0.23 (0.004–4.84)		96.2	96.2	none
Wang, 2005 [10]	35	brain	GK	14/21	66 (24–114)	17.2 (12–24)	N/A	94	71		none
Matsunaga, 2007 [11]	22	brain	GK	12/10	63 (9–146)	14 (8–30)	1.69 (0.0097–16.4)		88	78	none
Kano, 2008 [12]	32	brain	GK	19/13	50.1 (6.0–165.4)	16.0 (11–20)	0.72 (0.08–16.6)		91.9		none
Moss, 2009 [13]	31	brain and spine	Linac/CK	5/26	69(5–164)	23.4 (12–40)	1. (0.058–65.4)	85 (at 3 years)	82		5-radiation necrosis
Karabagli, 2010 [14]	13	brain	GK	6/7	50.2 (24–116)	15.8 (12–25)	0.022 (0.0005–11.37)	100			none
Sayer, 2011 [15]	14	brain	GK	7/7	36 (6–144)	18 (10–25)	1.65 (0.08–9.02)		74	50	none
Selch, 2012 [16]	9	spine	linac	4/5	51(14–86)	12	0.7 (0.08–14.4)		95 (at 4 years)		none
Our study	14	brain	linac/CK	5/9	24 (11–89)	20(10–32)	0.26 (0.026–20.4)	88%	73 (at 6 years)		none

GK = GammaKnife, VHL = Von-Hippel-Lindau disease, CK = CyberKnife, SIADH = syndrome of inappropriate antidiuretic hormone secretion, N/A = data not available.

### Factors associated with tumor control

#### Cystic lesions

In this study, there was a trend for tumors with a cystic component to progress. Two patients who had local failure enlargement of the cystic portion were treated with salvage surgery afterward. This is in agreement with most studies, which conclude that hemangioblastomas with large cysts are not suitable for SRS. The presence of a cystic component is significantly associated with poor LC [11–12]. Three other reports found that only the enhancing part, not the cystic portion of the contoured target volume, is effectively controlled by SRS [7, 10, 13]. The response of the cystic component to SRS, if any, was delayed. Most authors concluded that SRS should be reserved for small cystic hemangioblastomas or for patients with limited surgical options.

#### Tumor size/volume

Data from the literature have revealed that larger tumor volume is significantly associated with poorer progression-free survival after GammaKnife (GK) [7, 12], while another study only observed a trend in this direction [15]. In contrast, neither our study nor a report from Matsunaga *et al.* detected a correlation in the size of the tumor with growth control after SRS [11].

#### Solitary vs multiple tumors

Because of the paucity of available data, this particular aspect of tumors treated with SRS is not consistently described in the literature. Sayer *et al.* [15] found that multiple lesions were 7.9 times more prone to tumor progression, but this was in contrast to other reports [10, 11] and our study, which showed no significant difference in tumor control between solitary and multiple hemangioblastomas.

#### Von Hippel-Lindau disease

In the case series of VHL-associated hemangioblastomas, LC rates were 83–97%, 83% and 61% at 2, 5 and 10 years, respectively [17–20] (Table 4). Some previous reports noted worse progression-free survival for sporadic hemangioblastomas than for VHL-associated hemangioblastomas [12, 15] However, our study and that of Matsunaga *et al.* did not observe such a difference [11].

**Table 4.** Summary of the local control rates from previously reported series of VHL-associated intracranial hemangioblastoma treated with radiosurgery

author	No of pts	RT technology	Median F/U (range) (months)	Median dose (range) (Gy)	Median volume (range) (cm^3^)	2 year	5 year	10 year	Complication
Page, 1993 [17]	4	linac	17	35 (30–75)	N/A	100 (at 1 year)			none
Chang, 1998 [18]	13	linac	43 (11–84)	23.2 (18–40)	1.6 (0.07–65.4)	97			3-radiation necrosis
Rajaraman, 2004 [19]	14	GK	34 (10–58)	N/A	N/A	83			none
Asthagiri, 2010 [20]	20	linac/GK	101 (36–211.2)	18.9 (12–24)	0.5 (0.01–3.6)	91	83	61	none

GK = GammaKnife, VHL = Von-Hippel-Lindau disease, N/A = data not available.

#### Prescribed radiation dose

Until now, there has been no large case series (*n* > 100) with radiosurgery to standardize the generally accepted optimal dose for CNS hemangioblastoma. In most of the studies that reported a good outcome, this was commonly correlated with a high radiation dose [6, 7, 10]. The median marginal dose of 20 Gy used in our study was considered to be the proper prescribed dose for CNS HB, as reported previously [10].

The acute side effects of SRS were tolerable and could be managed on an outpatient basis. Our patients did not need any premedication before radiation. No late radiation complication was reported in this study. The lack of late radiation complications found in our study was not only a result of appropriate selection criteria for SRS treatment but also of some precautions that were implemented in the radiation technique. First, for small tumors (≤3 cm), we used optimal marginal radiation doses with a median dose of 20 Gy in a single fraction. For larger tumors (>3 cm), or tumors near critical organs such as the optic nerve or brainstem, it is generally accepted that the radiation dose to these structures should be limited to <10–12 Gy for a single-session treatment [21, 22]. If the single-session radiosurgery plan exposes critical structures beyond their tolerance doses as mentioned above, a fractionated course of 2–5 sessions is considered. Second, for X-Knife planning, we selected the optimal collimator size and the minimum number of isocenters able to cover the whole tumor. Third, we kept the treatment parameters such as comformity index and homogeneity index within the range of 1–2.

## CONCLUSION

In conclusion, linac-based SRS for intracranial hemangioblastoma is an effective and safe method of treatment. There was a trend toward local failure only in cystic tumors, but this trend was not found to be statistically significant. However, because of the relatively short-term follow-up of this study and the relatively small number of cases in the available literature, long-term clinical outcome is yet to be assessed for its sustainability.

## FUNDING

Funding to pay the Open Access publication charges for this article was provided by Research fund and data analysis unit, Ramathibodi Hospital, Mahidol University.
